# Model Fit after Pairwise Maximum Likelihood

**DOI:** 10.3389/fpsyg.2016.00528

**Published:** 2016-04-21

**Authors:** M. T. Barendse, R. Ligtvoet, M. E. Timmerman, F. J. Oort

**Affiliations:** ^1^Department of Data Analysis, Faculty of Psychology and Educational Sciences, Ghent UniversityGhent, Belgium; ^2^Department of Education, Research Institute of Child Development and Education, University of AmsterdamAmsterdam, Netherlands; ^3^Department Psychometrics and Statistics, Heymans Institute for Psychological Research, Psychometrics and Statistics, University of GroningenGroningen, Netherlands

**Keywords:** discrete data, pairwise maximum likelihood analysis, weighted least squares analysis, fit statistics

## Abstract

Maximum likelihood factor analysis of discrete data within the structural equation modeling framework rests on the assumption that the observed discrete responses are manifestations of underlying continuous scores that are normally distributed. As maximizing the likelihood of multivariate response patterns is computationally very intensive, the sum of the log–likelihoods of the bivariate response patterns is maximized instead. Little is yet known about how to assess model fit when the analysis is based on such a pairwise maximum likelihood (PML) of two–way contingency tables. We propose new fit criteria for the PML method and conduct a simulation study to evaluate their performance in model selection. With large sample sizes (500 or more), PML performs as well the robust weighted least squares analysis of polychoric correlations.

## 1. Introduction

Tests and questionnaires usually consist of items with discrete ordinal response scales. In the factor analysis of discrete item responses, multivariate normally distributed scores are assumed to underly the discrete item responses (e.g., Wirth and Edwards, 2007; Rhemtulla et al., 2012).

Let *X* = (*X*_1_, *X*_2_, …, *X*_*k*_) denote the vector of the *k* variables with discrete response scales, with realizations *x*_*i*_ ∈ {1, 2, …, *m*_*i*_}, so that each item *i* has *m*_*i*_ response options. The observed score *x*_*i*_ on item *i* is related to the unobserved score $x_{i}^{*}$ on the underlying continuum through

(1) $$\begin{array}{l} X_{i}=x_{i}\;\Leftrightarrow \;\tau_{x_{i}-1}<x_{i}^{*}\leq \tau_{x_{i}}, \end{array}$$

where τ_*x*_*i*_ − 1_ and τ_*x*_*i*__ are the threshold parameters for the category of item *i*. An item with *m*_*i*_ categories really only has *m*_*i*_ − 1 thresholds, as τ_0_ ⇒ − ∞ and τ_*m*_*i*__ ⇒ ∞. Herinafter, to simplify notation, we assume that the number of response options is equal across items, *m*_*i*_ = *m* for all *i*.

As the underlying continuous variable $X_{i}^{*}$ is not observed, its mean and variance are not identified without further constraints. One can either fix the mean and variance (e.g., zero mean and unit variance), or one can fix two of the thresholds (e.g., at zero and unity). The latter is not possible with dichotomous items, because they are associated with just a single threshold.

Various estimation methods have been proposed for the factor analysis of (observed) discrete responses with (unobserved) underlying continuous scores. Here we discuss the weighted least squares method, the multivariate maximum likelihood method, and the bivariate maximum likelihood method.

The *weighted least squares method* was introduced as a two–step method. In the first step, the polychoric correlations between the observed variables are estimated. In the second step, the parameters of the structural equation model are estimated on the basis of the polychoric correlations. The general WLS fit function for discrete data, based on Browne (1984) who described the WLS fit for continuous data, is given by

(2) $$\begin{array}{l} F_{WLS}={\left(\hat{q}-g\right)}^{\prime }W^{-1}\left(\hat{q}-g\right), \end{array}$$

where $\hat{q}$ is a vector with the non–redundant elements of the *k* × *k* matrix of polychoric correlations and *g* is a vector with the corresponding elements of the *k* × *k* matrix of model–implied correlations. The weight matrix *W* is a positive definite matrix of order *v* × *v*, with *v* = *k*(*k* + 1)∕2. It contains consistent estimates of the asymptotic variances and covariances of the polychoric correlations (e.g., Jöreskog, 1990, 1994). Other authors also included the observed and model implied threshold values in the $\hat{q}$ and *g* vectors, and the associated asymptotic covariances of $\hat{q}$ in matrix *W*, which resulted in two–step (see Lee et al., 1995) and three–step approaches (e.g., Muthén, 1984, 1989; Lee et al., 1990b). As the weight matrix can only be accurately estimated with large sample sizes (e.g., Rigdon and Ferguson, 1991; Muthén and Kaplan, 1992; Dolan, 1994), it is practically unfeasible to use the WLS function with the full weight matrix. An alternative is to use the WLS function with diagonal matrix *W*_*D*_, containing only the diagonal elements of *W* to estimate the parameter estimates. However, for inference, one needs the full weight matrix as implemented in the so–called robust WLS. The three–step robust WLS with mean–and–variance corrected chi-square and standard errors (WLSMV; Muthén et al., 1997; Asparouhov and Muthén, 2010), also referred to as RDWLS (see Katsikatsou et al., 2012), has been advocated because of good performance in simulation studies (e.g., Beauducel and Herzberg, 2006; Barendse et al., 2015).

In the *multivariate maximum likelihood estimation method* (Lee et al., 1990a), the maximum likelihood estimator is used to estimate the variances, covariances, means, and thresholds of all X^*^ simultaneously, in a single step. The method is also known as the full information maximum likelihood method, as one maximizes the likelihoods of the complete response patterns. This implies that one uses all information in the data, and does not have to rely on polychoric correlations, like in the WLS related estimation methods. Let ρ denote the vector containing the correlations between all pairs of continuous variables $X_{i}^{*}$ and $X_{j}^{*}$ with *i, j* = 1…*k*, and *i* < *j*. The expected proportion π of response vector *x*, given correlations ρ and thresholds τ, is given by

(3) $$\begin{array}{r} \pi_{x_{1},x_{2},\ldots ,x_{k}}\left(\rho ,\tau \right)\;=\;Pr\left(X_{1}=x_{1},X_{2}=x_{2},\ldots ,X_{k}=x_{k}|\rho ,\tau \right)\\ =\overset{\tau_{x_{1}}}{\underset{\tau_{x_{1}-1}}{\int }}\overset{\tau_{x_{2}}}{\underset{\tau_{x_{2}-1}}{\int }}\ldots \overset{\tau_{x_{k}}}{\underset{\tau_{x_{k}-1}}{\int }}f\left(x_{1}^{*},x_{2}^{*},\ldots ,x_{k}^{*}|\rho ,\tau \right)dx_{1}^{*}dx_{2}^{*}\ldots dx_{k}^{*}, \end{array}$$

where *f* denotes the *k*-dimensional normal density. Let index *r* refer to a complete item response pattern (*x*_1_, *x*_2_, …, *x*_*k*_), and let *p*_*r*_ denote the observed proportion of respondents with response pattern *r* in the sample. The log–likelihood of response pattern *r* is given by

(4) $$\begin{array}{r} \ln\;L\left(\rho ,\tau \right)=\overset{m^{k}}{\underset{r=1}{\sum }}p_{r}\ln\left[\pi_{r}\left(\rho ,\tau \right)\right]+constant, \end{array}$$

which is maximized to obtain the estimates for the parameters ρ and τ. As maximizing this log–likelihood requires numerical evaluation of high–dimensional integration over x^*^ (Equation 3) in order to obtain the probability function of a response vector, Jöreskog and Moustaki (2001) already concluded that FIML is only feasible with a small numbers of variables (e.g., four or less). This seriously limits the application of FIML in practice.

In the *bivariate maximum likelihood estimation method*, high numerical integration is avoided by considering bivariate information only. In this one–step method, the sum of the log–likelihoods of all possible bivariate response patterns is maximized, rather than that of the full multivariate response patterns.

For two items *i* and *j*, the expected proportion of respondents with scores *x*_*i*_, *x*_*j*_ is given by

(5) $$\begin{array}{r} \pi_{x_{i},x_{j}}\left(\rho_{ij},\tau_{i},\tau_{j}\right)=\overset{\tau_{x_{i}}}{\underset{\tau_{x_{i}-1}}{\int }}\overset{\tau_{x_{j}}}{\underset{\tau_{x_{j}-1}}{\int }}f\left(x_{i}^{*},x_{j}^{*}|\rho_{ij},\tau_{i},\tau_{j}\right)dx_{i}^{*}dx_{j}^{*}, \end{array}$$

for τ_*i*_ = (τ_1_*i*__, τ_2_*i*__, …, τ_*m*−1_) and τ_*j*_ = (τ_1_*j*__, τ_2_*j*__, …, τ_*m*−1_). In order to obtain the likelihood estimates of the parameters ρ_*ij*_ and τ_*i*_, τ_*j*_, instead of maximizing the multivariate likelihood, we maximize the sum of all bivariate log–likelihoods:

(6) $$\begin{array}{rcl} \ln\;L\left(\rho_{ij},\tau_{i},\tau_{j}\right) & = & \overset{k-1}{\underset{i=1}{\sum }}\overset{k}{\underset{j=i+1}{\sum }}\overset{m}{\underset{x_{i}=1}{\sum }}\overset{m}{\underset{x_{j}=1}{\sum }}p_{x_{i},x_{j}}\ln\left[\pi_{x_{i},x_{j}}\left(\rho_{ij},\tau_{i},\tau_{j}\right)\right]\\ +constant, \end{array}$$

where *p*_*x*_*i*_, *x*_*j*__ denotes the sample proportion of responses *x*_*i*_ and *x*_*j*_.

Jöreskog and Moustaki (2001) denoted this method the underlying bivariate normal method. They originally suggested to use both the univariate and bivariate distributions. Based on results of their simulation study (Katsikatsou et al., 2012) concluded that the univariate distributions have no additional value in the parameter estimation. The estimation method that only relies on bivariate likelihoods is referred to as the pairwise maximum likelihood (PML) method.

The PML estimation method has the advantage over FIML that it is computationally feasible, but it has the disadvantage that it only uses the bivariate distributions of the observed variables, and thus does not utilize all available information.

As an overall measure of fit, Jöreskog and Moustaki (2001) proposed to use the average of all bivariate likelihood ratio test statistics, but this statistic cannot be used as a goodness–of–fit test as its distribution is unknown. Maydeu-Olivares (2006) and Maydeu-Olivares and Joe (2006) introduced a family of fit statistics for testing composite null hypotheses in multidimensional contingency tables. As the PML method has been recognized as a special case of the maximum composite likelihood method (Varin, 2008; Varin et al., 2011) and the bivariate maximum likelihood estimation method, it can be used to obtain a residual based fit statistics (Maydeu-Olivares, 2006; Maydeu-Olivares and Joe, 2006) and standard errors (Xi, 2011) for the PML estimation method. In a simulation study, Xi (2011) found the composite likelihood fit statistic and standard errors estimates of the bivariate maximum likelihood estimation method to be appropriate, when compared to a full information expectation maximization algorithm. However, these test statistics are not yet readily available, as the have not yet been implemented in a computer program.

In the present paper, we propose three new fit statistics. We investigate these test statistics in a simulation study and compare them with the overall goodness–of–fit that is associated with robust WLS estimation. The new fit statistics have been made available in the open source SEM software lavaan (see Appendix in Supplementary materials; Rosseel, 2012).

## 2. Methods

To evaluate the three new fit statistics (explained in Section 2.2) for the PML estimation method, we conduct a simulation study in which we vary sample size (200, 500, and 1,000) and the number of response options (2, 3, and 4) in a fully crossed design, yielding nine conditions. With 1,000 replications, we obtain 9,000 datasets that are analyzed using the PML and robust WLS estimation methods.

### 2.1. Data generation

We partly replicate the simulation study conducted by Katsikatsou et al. (2012). They generated item scores on six items according to a two factor model with factor loadings

(7) $$\begin{array}{l} \Lambda =\left[\begin{array}{cc} 0.9 & 0\\ 0.8 & 0\\ 0.7 & 0\\ 0.5 & 0.6\\ 0 & 0.7\\ 0 & 0.8 \end{array}\right], \end{array}$$

common factor variances and covariances

(8) $$\begin{array}{l} \Phi =\left[\begin{array}{cc} 1 & 0.5\\ 0.5 & 1 \end{array}\right], \end{array}$$

and residual variances

(9) $$\begin{array}{l} \Theta =I-diag\left(\Lambda \Phi \Lambda^{\prime }\right). \end{array}$$

Continuous item scores are drawn from a multivariate normal distribution with variances and covariances

(10) $$\begin{array}{l} \Sigma =\Lambda \Phi \Lambda^{\prime }+\Theta , \end{array}$$

and zero means. For each sample size (200, 500, and 1000), we generate 1000 datasets of continuous scores. These scores are categorized into two categories (threshold 0, yielding expected proportions 0.50 and 0.50), three categories (thresholds –0.6 and 0.6, yielding expected proportions of 0.27, 0.45, and 0.27), and four categories (thresholds –1.2, 0, and 1.2, yielding expected proportions 0.11, 0.39, 0.39, and 0.11; in line with Katsikatsou et al., 2012).

### 2.2. Model fit statistics

In the PML method, model parameters are estimated by maximizing the sum of the log–likelihoods of all bivariate responses patterns, for all pairs of items. As the distribution of this sum is not known, we propose three measures of fit that are based on likelihood ratios: *C*_*F*_, *C*_*M*_, and *C*_*P*_. The *C*_*F*_ and *C*_*M*_ fit statistics compare the model–implied proportions of response patterns with, respectively, the observed proportions of full response patterns (signified by subscript *F*) and the expected proportions under the assumption of multivariate normality (signified by subscript *M*). The *C*_*P*_ fit statistic compares the model–implied proportions of pairs of item responses to the observed proportions of pairs of item responses (signified by subscript *P*).

Specifically, *C*_*F*_ compares the log–likelihood of the expected proportions of the multivariate response patterns (Equation 4) with the observed proportions of response patterns. Multiplied by two times the sample size, we obtain

(11) $$\begin{array}{l} C_{F}=2N\overset{m^{k}}{\underset{r=1}{\sum }}p_{r}\ln\left[p_{r}∕{\hat{\pi }}_{r}\right], \end{array}$$

that is asymptotically chi–square distributed with degrees of freedom equal to the difference between the number of possible response patterns and the number of model parameters to be estimated minus one (Agresti, 2002, pp. 590–591),

(12) $$\begin{array}{l} df_{F}=m^{k}-n-1, \end{array}$$

where *n* is the number of parameters to be estimated. As the number of possible response patterns *m*^*k*^ is usually much larger than sample size *N*, most response patterns will not be observed at all, yielding many empty cells in the multivariate *m*^*k*^ table, thereby causing bias in the *C*_*F*_ statistic. As a possible solution (Jöreskog and Moustaki, 2001) considered the number of response patterns that is actually observed only, and calculated degrees of freedom as

(13) $$\begin{array}{l} df_{F^{*}}=u_{r}-n-1, \end{array}$$

where *u*_*r*_ denotes the number of observed response patterns.

The fit statistic *C*_*M*_ compares the log–likelihood of the the model–implied proportions of response patterns of the model–of–interest with the model–implied proportions of the model that only assumes an underlying multivariate normal distribution (without any further restrictions):

(14) $$\begin{array}{l} C_{M}=C_{F1}-C_{F0}, \end{array}$$

where *C*_*F*1_ is *C*_*F*_ for Model 1, the model of interest, and *C*_*F*0_ is *C*_*F*_ for Model 0, the model that assumes underlying multivariate normality and that has all polychoric correlations ρ and all thresholds τ as its parameters. Statistic *C*_*M*_ has asymptotically a chi–square distribution with degrees of freedom equal to the difference in the numbers of parameters of Models 0 and 1,

(15) $$\begin{array}{l} df_{M}=k\left(k-1\right)∕2+k\left(m-1\right)-n_{1}, \end{array}$$

where *k*(*k* − 1)∕2 is the number of polychoric correlations, *k*(*m* − 1) is the number of thresholds, and *n*_1_ is the number of parameters of the model of interest. If the bias in *C*_*F*1_ and *C*_*F*0_ caused by empty cells in the *m*^*k*^ table cancels out in *C*_*M*_, then *C*_*M*_ may outperform *C*_*F*_.

The fit statistic *C*_*P*_ is based on pairs of responses only, by comparing the observed and model–implied proportions of those pairs. For items *i* and *j* (Agresti, 2002),

(16) $$\begin{array}{l} C_{P_{ij}}=2N\overset{m}{\underset{x_{i}=1}{\sum }}\overset{m}{\underset{x_{j}=1}{\sum }}p_{x_{i},x_{j}}\ln\left[p_{x_{i},x_{j}}∕{\hat{\pi }}_{x_{i},x_{j}}\right], \end{array}$$

which has an asymptotic chi–square distribution with degrees of freedom equal to the information (which is (*m*^2^ − 1)) minus the number of parameters [i.e., 2(*m*−1) thresholds and 1 correlation],

(17) $$\begin{array}{l} df_{P}=m^{2}-2m. \end{array}$$

To test the overall goodness-of-fit of the model, we consider all *C*_*P*_*ij*__ and select *C*_*P*_ = maximum (*C*_*P*_*ij*__). As there are *k*(*k* − 1)∕2 possible pairs of items, this *C*_*P*_ should be applied with a Bonferroni adjusted level of significance α^*^, with

(18) $$\begin{array}{l} \alpha^{*}=\frac{2\alpha }{k\left(k-1\right)}, \end{array}$$

to keep the family–wise error rate at α. The hypothesis of overall goodness-of-fit is tested at α and rejected when *C*_*P*_ is significant at α^*^. Notice that with dichotomous items, *m* = 2, *df*_*P*_ = 0, so that the hypothesis of an underlying bivariate normal distribution cannot be tested. So, statistic *C*_*P*_ can only be applied when there are more than two response options.

We will compare the performance of these statistics with the chi-square measure of overall goodness–of–fit that is associated with robust WLS estimation, which we will refer to as *C*_*W*_. To account for the violation of distributional assumptions, this *C*_*W*_ statistic is subject to a scaling correction (Muthén et al., 1997; Satorra and Bentler, 2001; Asparouhov and Muthén, 2010). Here we will use the mean–and–variance corrected chi–square statistic (Asparouhov and Muthén, 2010).

### 2.3. Analysis

We fit three models to each of the 9,000 datasets: a baseline model, a one–factor model, and a two–factor model. The baseline model includes all polychoric correlations and thresholds. If the baseline model does not fit then we must reject the hypothesis of an underlying multivariate normal distribution. The one–factor model has a free 6 × 1 matrix Λ and the 1 × 1 matrix Φ is fixed at unity. The two-factor model corresponds to the data generation model and has a 6 × 2 matrix Λ with a pattern of free factor loadings that corresponds with Λ above, a 2 × 2 symmetric matrix Φ with diagonal elements fixed at unity and a free off–diagonal element. In both the one–factor and two–factor model Θ is a 6 × 6 diagonal matrix equal to *I*−*diag*(Λ*ΦΛ*′).

We use two estimation methods: PML and robust WLS. Model fit will be evaluated with measures *C*_*F*_, *C*_*M*_, and *C*_*P*_ after PML estimation and with measure *C*_*W*_ after robust WLS estimation. The computer program Mx (Neale et al., 2002) is used for PML estimation, and the computer program Mplus 6.11 (Muthén and Muthén, 2010) for robust WLS estimation. The computer program R is used to calculate the fit measures *C*_*F*_, *C*_*M*_, and *C*_*P*_ (using the “mvtnorm” package; R version 2.12.0; R Development Core Team, 2010).

The performance of the four fit measures will be evaluated by calculating the proportions of model rejection in each of the conditions. The baseline model and the two–factor model should fit. When testing at a 5% level of significance, these two models should be rejected in 5% of all cases. The one–factor model should not fit and should always be rejected.

## 3. Results

Before presenting the results of the different methods for the evaluation of model fit, we briefly comment on the accuracy and efficiency of parameter estimation through PML. The accuracy is evaluated by calculating the absolute differences between the parameter estimates and the population values. The standard deviations indicate the efficiency of the parameter estimates.

Across all conditions, the average absolute difference of the factor loadings is 0.001 and the average standard deviation is 0.052. The average absolute difference of the correlation between the latent variables across all conditions is 0.002 and the standard deviation across all conditions is 0.069. Noteworthy, PML shows a slightly higher accuracy than robust WLS in terms of the estimates of the factor loadings and the correlation, with average absolute differences of 0.001 for PML and 0.003 for robust WLS. The efficiency is about the same. Katsikatsou et al. (2012) already reported on the accuracy and efficiency of the parameter estimates in the case of four point response options. Our results are consistent with the results of Katsikatsou et al. (2012).

### 3.1. The *C*_*F*_ fit statistic

Table 1 gives the results of fit evaluation with the *C*_*F*_ statistic for the baseline model, the one–factor model, and the two–factor model. For each condition, the means and standard deviations of the fit statistic are calculated across 1,000 replications. Rejection rates and 95% confidence intervals are given two times: Once with degrees of freedom based on the number of possible response patterns (*df*_*F*_, Equation 12) and with degrees of freedom based on the number of observed response patterns ($df_{F^{*}}$, Equation 13). Means and standard deviations of $df_{F^{*}}$ are given as well.

**Table 1:** ***C*_*F*_ Rejection rates**.

**Conditions**		***C***_*****F*****_		***C*_*F*_ with *df*_*F*_**				*C*_*F*_ with *df*_F^*^_				
**N**	**Scale**	**M(*C*_*F*_)**	**SD(*C*_*F*_)**	***df***	**RR**	**95% CI**		**M(*df*)**	**SD(*df*)**	**RR**	**95% CI**	
						***Q*_2.5_**	***Q*_97.5_**				***Q*_2.5_**	***Q*_97.5_**
**BASELINE MODEL**												
200	2-point	48.079	8.584	42	0.135	0.114	0.156	25.099	2.827	0.838	0.815	0.861
	3-point	292.774	21.491	701	0.000	-	-	91.058	5.436	1.000	-	-
	4-point	495.966	29.052	4,062	0.000	-	-	114.035	5.493	1.000	-	-
500	2-point	47.921	10.052	42	0.154	0.132	0.176	36.733	1.918	0.356	0.326	0.386
	3-point	396.944	25.581	701	0.000	-	-	173.271	7.140	1.000	-	-
	4-point	757.367	37.709	4,062	0.000	-	-	249.046	8.657	1.000	-	-
1000	2-point	44.524	9.815	42	0.088	0.070	0.106	40.354	0.786	0.139	0.118	0.160
	3-point	470.109	27.018	701	0.000	-	-	243.532	8.069	1.000	-	-
	4-point	982.595	41.825	4,062	0.000	-	-	389.423	10.847	1.000	-	-
**ONE-FACTOR MODEL**												
200	2-point	90.359	15.587	51	0.919	0.902	0.936	34.099	2.827	0.999	0.997	1.000
	3-point	363.119	25.975	710	0.000	-	-	100.058	5.436	1.000	-	-
	4-point	580.380	33.606	4,071	0.000	-	-	123.035	5.493	1.000	-	-
500	2-point	140.112	23.840	51	1.000	-	-	45.733	1.918	1.000	-	-
	3-point	561.390	34.551	710	0.000	-	-	182.271	7.140	1.000	-	-
	4-point	957.356	46.486	4,071	0.000	-	-	258.046	8.657	1.000	-	-
1000	2-point	221.741	31.113	51	1.000	-	-	49.354	0.786	1.000	-	-
	3-point	790.216	45.708	710	0.633	0.603	0.663	252.532	8.069	1.000	-	-
	4-point	1,374.973	59.976	4,071	0.000	-	-	396.423	10.847	1.000	-	-
**TWO-FACTOR MODEL**												
200	2-point	55.817	9.096	49	0.120	0.100	0.140	32.099	2.827	0.796	0.771	0.821
	3-point	300.436	21.524	708	0.000	-	-	98.058	5.436	1.000	-	-
	4-point	503.774	29.109	4,069	0.000	-	-	121.035	5.493	1.000	-	-
500	2-point	55.420	10.588	49	0.160	0.137	0.183	43.733	1.918	0.332	0.303	0.361
	3-point	404.481	25.652	708	0.000	-	-	180.271	7.140	1.000	-	-
	4-point	765.212	37.649	4,069	0.000	-	-	256.046	8.657	1.000	-	-
1000	2-point	52.086	10.773	49	0.096	0.078	0.114	47.354	0.786	0.135	0.114	0.156
	3-point	477.726	27.308	708	0.000	-	-	250.532	8.069	1.000	-	-
	4-point	990.351	41.933	4,069	0.000	-	-	398.423	10.847	1.000	-	-

*Means (M) and standard deviations (SD) of the fit statistic, rejection rates (RR) at a 5% level of significance, and 95% confidence intervals (CI) of the rejection rates are calculated across the 1000 simulated datasets*.

The fit of the baseline model is a test of the assumption of underlying multivariate normality, so we would expect rejection rates that equal the level of significance (5%). The overall rejection rates with the *df* based on the number of possible response patterns (*df*_*F*_) in conditions with two-point response scales are too high (13.5%, 15.4%, 8.8%). With three-point and four–point response scales, *df*_*F*_ is very large so that the baseline model never gets rejected. The same is true for the two–factor model that should fit the data, but is rejected too often in the conditions with two-point response scales (12.0%, 16.0%, 9.6%) and never rejected in the other conditions. The one-factor model is not correct and should be rejected, which is the case in conditions with two-point response scales but not in conditions with three-point scales and four-point scales.

We attribute the bad results in conditions with three-point scales and four-point scales to the large numbers of empty cells in the multivariate contingency tables. In the cases of three-point response scales and four-point response scales the numbers of possible response patterns are 729 and 4096, whereas the total numbers of observations are only 200, 500, or 1000, rendering the *C*_*F*_ statistic unsuitable. The overall rejection rates of the baseline model with degrees of freedom based on the number of observed response patterns (i.e., $df_{F^{*}}$) are consistently much too high, in all conditions, showing that the use of $df_{F^{*}}$ is not justified.

### 3.2. The *C*_*M*_ fit statistic

Table 2 gives the results of fit evaluation with the *C*_*M*_ statistic for the one–factor model and the two–factor model. The one–factor model is almost always rejected, except in the condition with sample size 200 and two–point response scales (with 0.987 rejection rate). The rejection rates for the two–factor model should be about equal to the level of significance (5%), but vary from 6.8% to 9.6%.

**Table 2:** ***C*_*M*_ Rejection Rates**.

**Conditions**		***C***_*****M*****_		***df***	**RR (*C*_*M*_)**	**95% CI**	
**N**	**Scale**	**M(*C*_*M*_)**	**SD(*C*_*M*_)**			***Q*_2.5_**	***Q*_97.5_**
**ONE–FACTOR MODEL**							
200	2-point	42.280	13.885	9	0.987	0.980	0.994
	3-point	70.344	18.109	9	1.000	-	-
	4-point	84.415	21.178	9	1.000	-	-
500	2-point	92.191	20.965	9	1.000	-	-
	3-point	164.446	28.851	9	1.000	-	-
	4-point	199.989	31.577	9	1.000	-	-
1000	2-point	177.217	28.776	9	1.000	-	-
	3-point	320.107	40.680	9	1.000	-	-
	4-point	392.379	43.946	9	1.000	-	-
**TWO–FACTOR MODEL**							
200	2-point	7.738	4.260	7	0.091	0.073	0.109
	3-point	7.661	4.099	7	0.072	0.056	0.088
	4-point	7.808	4.173	7	0.083	0.066	0.100
500	2-point	7.499	3.870	7	0.068	0.052	0.084
	3-point	7.537	4.170	7	0.081	0.064	0.098
	4-point	7.845	4.387	7	0.096	0.078	0.114
1000	2-point	7.561	4.143	7	0.085	0.068	0.102
	3-point	7.617	4.164	7	0.082	0.065	0.099
	4-point	7.756	3.958	7	0.080	0.063	0.097

*Means (M) and standard deviations (SD) of the fit statistic, rejection rates (RR) at a 5% level of significance, and 95% confidence intervals (CI) of the rejection rates are calculated across the 1000 simulated datasets*.

Overall, we consider the *C*_*M*_ results satisfactory. Apparently, the sparseness of data and (almost) empty cells that invalidate the use of the *C*_*F*_ statistic does not seem to affect the *C*_*M*_ statistic much.

### 3.3. The *C*_*P*_ fit statistic

The *C*_*P*_ results are given in Table 3. As explained above, the *C*_*P*_ statistic cannot be used with two-point response scales. For all other conditions Table 3 gives the means, standard deviations, and rejection rates of the highest *C*_*P*_ among the 15 bivariate tests that are conducted with each dataset. To guard against inflation of the family–wise error rate, the level of significance is adjusted to 5% / 15 = 0.33%.

**Table 3:** ***C*_*P*_ Rejection rates**.

**Conditions**		***C***_*****P*****_		***df***	**RR (*C*_*P*_)**	**95% CI**	
**N**	**Scale**	**M(*C*_*P*_)**	**SD(*C*_*P*_)**			***Q*_2.5_**	***Q*_97.5_**
**BASELINE MODEL**							
200	3-point	8.653	2.783	3	0.050	0.036	0.064
	4-point	16.044	3.656	8	0.046	0.033	0.059
500	3-point	8.393	2.893	3	0.055	0.041	0.069
	4-point	15.953	3.740	8	0.050	0.036	0.064
1000	3-point	8.419	2.846	3	0.049	0.036	0.062
	4-point	16.141	3.640	8	0.044	0.031	0.057
**ONE–FACTOR MODEL**							
200	3-point	16.577	5.307	3	0.670	0.641	0.699
	4-point	24.338	6.159	8	0.539	0.508	0.570
500	3-point	31.554	9.173	3	0.996	0.992	1.000
	4-point	42.918	10.273	8	0.995	0.991	0.999
1000	3-point	58.083	12.343	3	1.000	-	-
	4-point	76.562	14.507	8	1.000	-	-
**TWO–FACTOR MODEL**							
200	3-point	8.918	2.789	3	0.057	0.043	0.071
	4-point	16.306	3.675	8	0.052	0.038	0.066
500	3-point	8.626	2.908	3	0.060	0.045	0.075
	4-point	16.190	3.744	8	0.049	0.036	0.062
1000	3-point	8.672	2.844	3	0.054	0.040	0.068
	4-point	16.409	3.659	8	0.054	0.040	0.068

*Means (M) and standard deviations (SD) of the fit statistic, rejection rates (RR) at a 5% level of significance, and 95% confidence intervals (CI) of the rejection rates are calculated across the 1000 simulated datasets*.

The rejection rates for the baseline model vary between 4.4% and 5.5%, and for the two–factor model between 3.5% and 6.0%, which is reasonably close to the significance level of 5%. The one–factor model is almost always rejected in conditions with sample sizes of 500 and 1,000. However, in the small sample conditions rejection rates are only 67.0% and 53.9%.

### 3.4. The *C*_*W*_ fit statistic

For the purpose of comparison, Table 4 gives the *C*_*W*_ results after analysing all data sets with the robust WLS method of estimation. The one-factor model is almost always rejected. The rejection rates for the two-factor model vary between 3.9% and 6.4%.

**Table 4:** ***C*_*W*_ Rejection rates**.

**Conditions**		***C***_*****W*****_		***df***	**RR (*C*_*W*_)**	**95% CI**	
**N**	**Scale**	**M(*C*_*W*_)**	**SD(*C*_*W*_)**			***Q*_2.5_**	***Q*_97.5_**
**ONE–FACTOR MODEL**							
200	2-point	46.014	14.775	9	0.994	0.989	0.999
	3-point	73.982	19.193	9	1.000	-	-
	4-point	88.832	22.784	9	1.000	-	-
500	2-point	102.986	23.243	9	1.000	-	-
	3-point	177.773	30.827	9	1.000	-	-
	4-point	213.134	34.428	9	1.000	-	-
1000	2-point	201.828	33.761	9	1.000	-	-
	3-point	348.523	43.996	9	1.000	-	-
	4-point	421.149	47.477	9	1.000	-	-
**TWO–FACTOR MODEL**							
200	2-point	7.032	3.780	7	0.044	0.031	0.057
	3-point	6.933	3.557	7	0.041	0.029	0.053
	4-point	6.992	3.686	7	0.060	0.045	0.075
500	2-point	7.028	3.557	7	0.053	0.039	0.067
	3-point	6.804	3.604	7	0.039	0.027	0.051
	4-point	7.186	3.530	7	0.044	0.031	0.057
1000	2-point	7.114	3.967	7	0.064	0.049	0.079
	3-point	7.056	3.803	7	0.054	0.040	0.068
	4-point	7.012	3.548	7	0.046	0.033	0.059

*Means (M) and standard deviations (SD) of the fit statistic, rejection rates (RR) at a 5% level of significance, and 95% confidence intervals (CI) of the rejection rates are calculated across the 1000 simulated datasets*.

The *C*_*W*_ results with the two-factor model are somewhat better (closer to 5% rejection rates) than the *C*_*M*_ results. The *C*_*W*_ results are about similar to the *C*_*P*_ results, except for the rejection rates of the one-factor model in small sample size conditions, in which the *C*_*W*_ statistic seems to have more power.

## 4. Discussion

We proposed three new statistics for goodness of overall fit of models that are fitted through the pairwise maximum likelihood (PML) method. With the *C*_*F*_ statistic we test the difference between the model–implied proportions of multivariate response patterns and the observed proportions of multivariate response patterns. With the *C*_*M*_ statistic we test the difference between model–implied proportions of multivariate response patterns and the proportions of response patterns that are implied by the assumption of underlying multivariate normally distributed continuous variables. With the *C*_*P*_ statistic we test the difference between model–implied proportions of bivariate response patterns and observed proportions of bivariate response patterns.

The *C*_*F*_ statistic appeared unsuitable for the evaluation of model fit. The performance of the *C*_*M*_ statistic was good, although the rejection rates for the two factor model were consistently a little too high (varying between 6.8% and 9.6% instead of 5%). The *C*_*P*_ statistic showed the best results with rejection rates close to the expected values (around 5% for models that should fit, and close to 100% for models that should not fit), except for relatively small sample sizes of 200 with which the rejection rates for the wrong one–factor model were substantially too low. For all fit statistics, we only reported results of testing at the 5% level of significance, as the results at the 1% level of significance were very similar.

As an aside, we note that in the condition with four response options and sample size 500, we have reported the results of a second drawing of 1,000 datasets. The first drawing produced by chance unexpected low *C*_*P*_ rejection rates for the baseline model (i.e., 0.032 with a confidence interval of 0.021–0.043) and one–factor model (i.e., 0.035 with a confidence interval of 0.025–0.046) that did not seem representative. No other statistics were affected.

The performance of the PML fit statistics is only partly dependent on sample size. The *C*_*F*_ statistic is not suitable with any sample size, as we observe the negative consequences of very large contingency tables affected by sparseness of data (e.g., Agresti and Yang, 1987; Reiser and VandenBerg, 1994; Reiser and Lin, 1999; Jöreskog and Moustaki, 2001; Bartholomew and Leung, 2002). The alternative way of calculating degrees of freedom of Jöreskog and Moustaki (2001) on the basis of the number of observed response patterns instead of the number of possible response patterns, appeared unsuitable. In practice, one can deal with sparseness by for example combining cells, reducing the number of categories, or eliminating the most offending variables (see Agresti and Yang, 1987; Jöreskog and Moustaki, 2001). However, it was not possible to implement this in this simulation study.

The *C*_*M*_ statistic seems not that much affected by sparseness of data. The *C*_*P*_ statistic uses bivariate tables only, but its power for rejecting the one–factor model is mediocre when the sample size is small. Still, the *C*_*P*_ rejection rates for the correct models are not affected by small sample size. In our simulation study we also varied the number of response options, but this manipulation did not affect the results of the *C*_*M*_ and *C*_*P*_ fit statistics much.

We compared the results of the PML fit statistics results with results of robust weighted least squares (WLS) with the adjusted chi–square statistic *C*_*W*_. The performance of *C*_*W*_ was very similar to the performance of *C*_*P*_, and in small sample conditions *C*_*W*_ outperformed *C*_*P*_ in rejecting the one–factor model. Still, robust WLS estimation is very different from PML estimation. Robust WLS is a multiple–step method that relies on the estimated polychoric correlations. The model–implied correlations are then fitted to fixed polychoric correlations, so there is no direct relation between the model–implied correlations and the observed discrete responses. That is why we really expected PML to behave better than robust WLS. However, in the present simulation study of six variables measuring two common factors, robust WLS did at least as well as PML.

We still do not know how robust WLS and PML compare in larger datasets, with more variables, and more complex models. As WLS relies on a two-step procedure in which summary statistics are calculated first, we would expect the single-step PML procedure to outperform the WLS procedure. PML may also show advantages over WLS in case of incomplete data. Finally, we think that the PML method is a feasible alternative to FIML in case of larger data sets. Overall, the PML method seems a promising method that can be used to estimate all structural equation models, such as exploratory factor analysis models, multigroup models and longitudinal models (Moustaki, 2003; Vasdekis et al., 2012). We used Mx to apply the PML method, but the PML fit estimates can also be obtained with OpenMX (Boker et al., 2011) and lavaan (Rosseel, 2012). To facilitate their use, the *C*_*F*_, *C*_*M*_, and *C*_*P*_ statistics have been implemented in lavaan (see Appendix in Supplementary materials; Rosseel, 2012).

## Author contributions

All authors meet the criteria for authorship. All authors contributed substantially to the conception and design of the work, and drafting and finalizing the paper. MB, RL, and FO designed and programmed the simulation study.

### Conflict of interest statement

The authors declare that the research was conducted in the absence of any commercial or financial relationships that could be construed as a potential conflict of interest.
