# Supplementary material for: Model Fit after Pairwise Maximum Likelihood
Source: Front Psychol. 2016 Apr 21;7:528. doi: 10.3389/fpsyg.2016.00528 (PMC4838635; doi:10.3389/fpsyg.2016.00528)
Supplement: Supplementary file 1 [file DataSheet1.pdf]

APPENDIX; LAVAAN CODE FOR C_F, C_M, AND C_P FIT STATISTICS

```
library(lavaan)

# Example of PML with CF, CM, and CP
HS9 <- HolzingerSwineford1939[,c("x1","x2","x3","x4","x5",
       "x6","x7","x8","x9")]
HSbinary <- as.data.frame( lapply(HS9, cut, 2, labels=FALSE) )

# Single group example with one latent factor
HS.model <- ' trait =~ x1 + x2 + x3 + x4 '
fit <- cfa(HS.model, data=HSbinary[,1:4], ordered=names(HSbinary),
       estimator="PML")

# fit statistics
lavTablesFitCm(fit)
lavTablesFitCp(fit)
lavTablesFitCf(fit)

# see https://github.com/yrosseel/lavaan/blob/master/R/ctr_pairwise_fit.R
```
